# Vigilance behaviors and EEG activity in sustained attention may affect acute pain

**DOI:** 10.15761/jsin.1000184

**Published:** 2017-12-02

**Authors:** JH Chien, A Korzeniewska, AE Hillis, JH Kim, N Emerson, JD Greenspan, CM Campbell, TJ Meeker, TM Markman, FA Lenz

**Affiliations:** 1 Department of Neurosurgery, Johns Hopkins University, Baltimore, USA; 2 Department of Neurology, Johns Hopkins University, Baltimore, USA; 3 Department of Neurosurgery, Korea University Guro Hospital, Seoul, Korea; 4 Department of Pain and Neural Sciences, Dental School, University of Maryland, Baltimore, USA; 5 Department of Psychiatry and Behavioral Sciences, Johns Hopkins University, Baltimore, USA; 6 Institute of Biomedical Engineering – Nanomedicine, National Health Research Institutions, Taiwan, Republic of China

**Keywords:** sustained attention, cortex, event related synchronization, scalp EEG, human, pain

## Abstract

During Sustained Attention to stimuli across many modalities neural activity often decreases over time on task, while Errors in task performance increase (Vigilance Decrement). Sustained Attention to pain has rarely been investigated experimentally despite its clinical significance. We have employed a Sustained Attention protocol (Continuous Performance Task, CPT) in which the subject counts painful laser stimuli (targets) when they occur randomly in a prolonged train of nonpainful nontargets. We hypothesize that the magnitude of the poststimulus oscillatory power divided by baseline power (Event-Related Spectral Perturbation, ERSP - scalp EEG) over Frontoparietal structures will decrease at all frequencies with time on task, while Beta ERSP (14-30Hz) will be correlated with Error Rates in performance of the CPT.

During the CPT with a painful target ERSP was found in four separate Windows, as defined by both their frequency band and the time after the stimulus. A Vigilance Decrement was found which confirms that Sustained Attention to pain was produced by this CPT. In addition, Error Rates was correlated inversely with laser energy, and with ratings of pain unpleasantness and salience. Error Rates also were related directly to the Beta ERSP Window at scalp EEG electrodes over the central sulcus. Over time on task, the ERSP magnitude decreased in Alpha (8-14Hz) Window, was unchanged in early and late Delta/Theta Windows (0-8Hz), and increased in the Beta Window. The increase in Beta ERSP and a decrease in the Alpha ERSP occurred at the same EEG electrode over the parietal lobe to a significant degree across subjects.

Overall, Beta activity increases with time on task, and with higher Error Rates as in the case of other modalities. In the case of pain increased Errors correspond to misidentification of painful and nonpainful stimuli and so modulate the sensation of pain under the influence of Sustained Attention.

## Introduction

Sustained attention toward pain is an adaptive mechanism, but when it is abnormally increased it is referred to as hyper vigilance, ‘excessive readiness to respond to a certain kind of stimulus’ [1], which can occur in chronic pain [2,3]. Experimental Sustained Attention can be produced by Continuous Performance Tasks (CPTs) in which the subject responds to target stimuli occurring in a prolonged train of nontarget stimuli [4]. Frontal Parietal Bloodflow and BOLD activity often decreases in CPTs with somatic, visual and auditory targets [5], while CPTs with painful targets have not been studied, to our knowledge.

Oscillatory power after a stimulus (ERSP) may decrease (ERD – Event-Related Desynchronization) or increase (ERS – Event-Related Synchronization) [6]. ERD and ERS occur in different frequencies following painful laser stimuli and in Alpha and Beta Windows during attention to painful stimuli [7-9].

Errors in CPTs can be related to Parietal Frontal Blood flow and BOLD signals [10], and to increased Beta power and synchrony [11]. Errors are related to the unpleasantness of an intense but nonpainful somatic target stimulus presented in a CPT [12], and to ongoing pain of any etiology in subjects during an online visual Sustained Attention task [13]. The occurrence, symptoms and response to treatment of some chronic pain syndromes have been related to errors in CPTs with visual targets [14-16]. We now test the hypothesis that painful target stimuli will induce changes in Frontal Parietal ERSP across all frequency bands over time on , and Beta ERSP will correlate with performance Errors.

## Methods

Sixteen healthy participants (10 men and 6 women; aged 22-57 years) were recruited in this study. The protocol for this study was approved and reviewed yearly by an Institutional Review Board of the Johns Hopkins University School of Medicine, and all participants signed an informed consent form prior to participation in this study. These data have been used in a previous study of directed attention but not Sustained Attention [7].

### Stimuli and experimental design

The laser and electrical stimuli were delivered by procedures which are standard in our lab [7] to produce sensations in the distribution of the superficial radial nerve. The laser stimuli (Neurotest, Wavelight, Starnberg, Germany, wavelength of 2 μm, diameter 6 mm, duration 1 ms) were applied to slightly different locations for each stimulus in order to avoid sensitization or fatigue of nociceptors [17]. The electrical stimulus was a single, square-wave pulse of constant current (Grass S12 Isolated Biphasic Stimulator, 1 ms), which was delivered through cutaneous electrodes (0.5 cm diameter, 1 cm inter-electrode distance) on the dorsum of the left wrist.

Subjects rated the pain intensity of the laser, as well as the unpleasantness and salience of both the laser and electrical stimuli on numerical rating scales from 0 to 10. The anchors for these scales were the absence of pain (or unpleasantness) and the greatest pain intensity (or unpleasantness) imaginable. Salience is a property of both painful and nonpainful stimuli [18,19], and is defined as ‘the ability of the stimulus to capture attention.’ It was rated on a scale anchored by 0 as the absence of salience and 10 as the most salient stimulus imaginable.

During testing before the CPT protocol, the laser energy was set to the level that evoked a pain intensity of 4-6/10, which resulted in an average laser energy level of 730 ± 170 mJ (mean +/− SD), and a pin prick sensation for all subjects. The electrical stimulus was set to the voltage that produced a salience rating equal to that of the laser stimulus in each subject, which resulted in an average voltage of 12.8 +/− 5.2 mV, and evoking a nonpainful tingling sensation in each subject. At these settings, baseline ratings for the electrical stimulus included the unpleasantness (2.3 +/− 1.3), and salience (5.3 +/− 1.9), while the laser stimulus ratings included pain intensity (5.1 +/− 0.8), unpleasantness (5.1 +/− 1.25), and salience (5.3 +/− 1.9) [7]. These intensities of electric and laser stimuli were applied throughout the CPT protocol.

Sustained Attention has been produced by tasks in which a subject responds to target stimuli by counting them or by a button press [4,20-22]. We elected to use a counting task in which participants were instructed to count either the laser or the electric target stimulus in different trains of stimuli in the protocol. In this protocol, the time on task consisted of two contiguous blocks in which the subjects counted laser pulses and two contiguous blocks in which they counted electrical stimuli. Each block was composed of approximately 40 electrical and 40 laser stimuli that were presented with random order and random interstimulus intervals (7 to 8 seconds). The task of the first two blocks (Blocks 1 and 2) was randomly assigned to either the count laser task or count electric task and counterbalanced across subjects, while the Blocks 3 and 4 were assigned to the task not assigned in Blocks 1 and 2.

Experimental Blocks 1 and 2 were designated as the First and Second Intervals during the analysis of the results [21,23]. Behavioral ratings and ERSP activity for the First versus the Second Interval were interpreted as indicators of the effect of time on task during Sustained Attention. Following each block, participants reported the number of target stimuli in the block and rated pain intensity, unpleasantness and salience of the laser. The Error Rate was defined as the number of Target stimuli counted divided by the number of Target Stimuli presented, and so is a parametric variable.

### EEG Event-Related Spectral Power (ERSP) analysis

EEG signals were recorded using Ag-AgCl electrodes (Grass) placed on the scalp at 19 electrode locations according to the international 10-20 system with a referential montage to linked earlobes. Signals were amplified and digitized at the sampling rate of 500 Hz (SynAmps 5083, Neuroscan). The timing for the onset of the laser and electrical stimuli were acquired and digitally embedded with the recordings.

In this study, we measured the event-related non phase-locked responses induced by the laser stimuli [24]. This technique measures significant event-related changes in the power spectrum across different frequency bands (Table 1) over time after the stimulus. In order to detect power changes across different frequencies, each poststimulus spectral power estimate was divided by the mean baseline power (see below) and this ratio was the ERSP.

Using the Fast Fourier Transform (FFT) approach, the analysis estimated the significant event-related changes in the amplitude of the power spectrum across different times and frequencies, as described below [7,24]. The FFTs were calculated over a period of 512 ms that was advanced by 18 ms steps throughout the whole epoch. The final time frequency matrix for each epoch was set to have 51 linear-spaced frequencies from 2 Hz to 99.6 Hz and 200 time stamps.

The analysis of the power spectrum was carried out by filtering at 0.1 to 250 Hz using a Hanning finite impulse response filter. The event-related EEG epochs were extracted from every trial from 0.5 s before and 2 s after the onset of the stimulus. All EEG epochs were visually inspected separately by two individuals for rejection of eye blink and movement related artifacts; fewer than 10 trials were rejected for each block. The time-frequency analysis was performed partly using *newtimf.m* in the EEGLAB, running in the 64-bit MATLAB (R2012a. 7.14.0.739 environment) [24].

**Figure 1.**
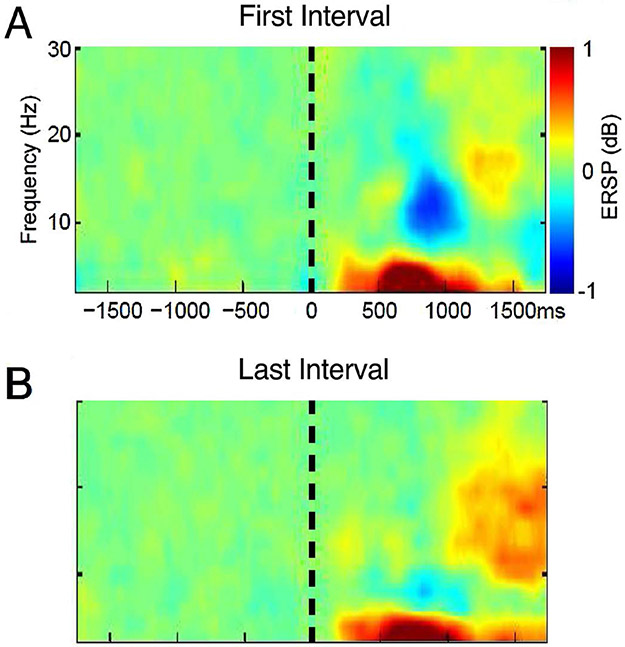
Time frequency plots of ERSP in response to the painful laser stimulus averaged across Subjects and Channels **A.** during the laser stimulus and count laser task in the First Interval of the CPT where the roman numerals indicate the location of Windows I to IV, and **B.** during count laser in the Second Interval.

**Figure 2.**
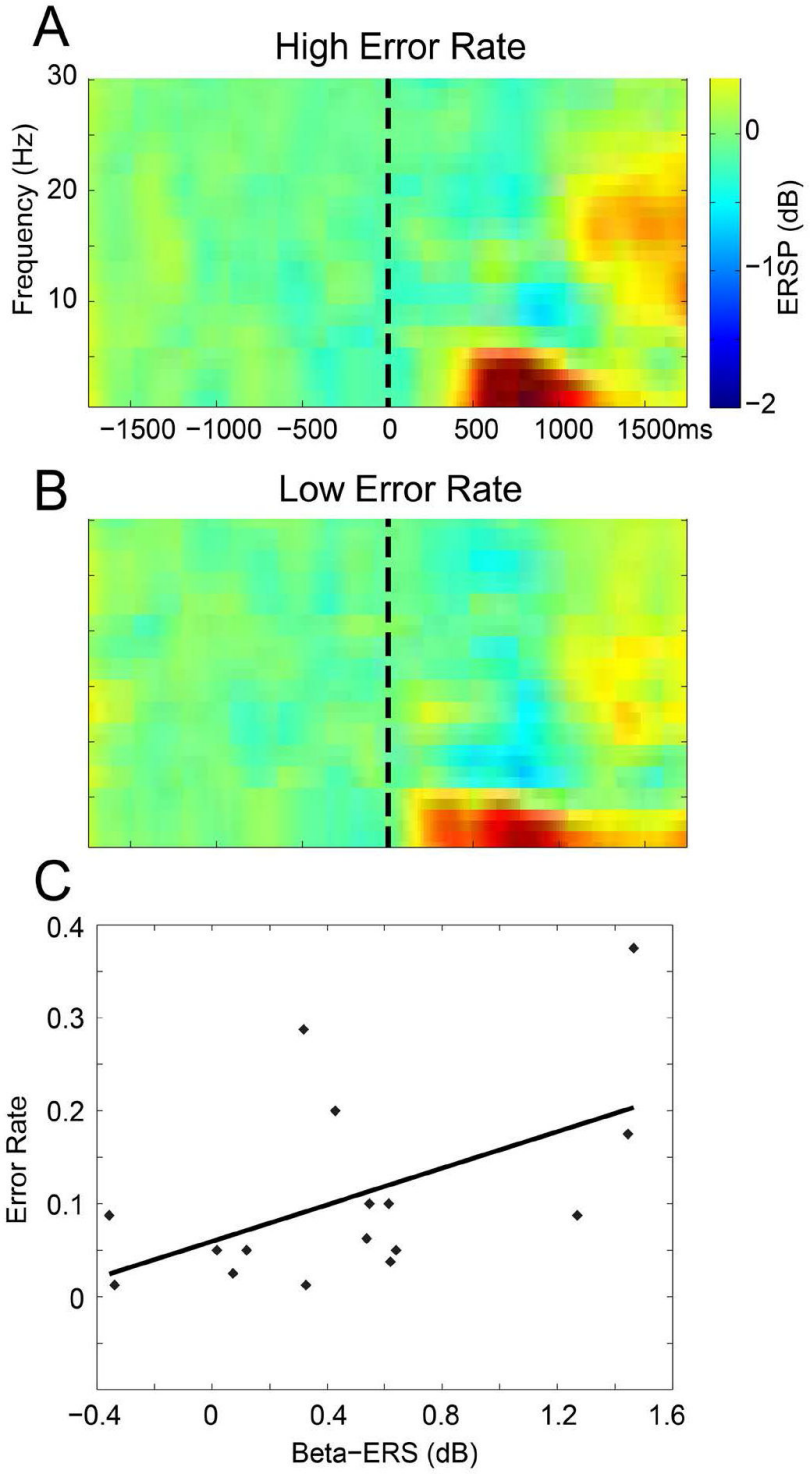
ERSP as a function of Error Rates: **A.** ERSP during a CPT with a painful target for subjects in the upper half of Error Rates for our population, and **B**. in the lower half of Error Rates; **C.** Error Rates versus Beta ERS (in dB) see text.

To establish the upper and lower ERSP thresholds for significance after the FFT application for each frequency step (from 2 to 99.6 Hz, step size 1.95 Hz) a bootstrap procedure (EEGLAB, ‘bootstat.m’) was performed. The spectral estimates in the trial and time dimensions were randomly resampled one thousand times during the baseline period of 0 to 0.5 s prestimulus [7,24]. The baseline in this approach includes approximately equal numbers cases in which a laser or an electrical stimulus precedes any trial so that the preceding trial does not bias the results. The final result of this analysis was presented as a ratio of the post-stimulus spectral power estimate over the baseline (ERSP). ERSP included both ERS (Event-Related Synchronization) if this ratio (in decibels, dB) was larger than 1 (Hot colors in Figures 1 and 2), and ERD (Event-Related Desynchronization) if it was less than 1 (Cold colors), and both were displayed relative to (Green) thresholds for significance. The selection of time frequency Windows (Table 1) was arbitrary based upon the overall results (Figure 1) without accounting for differences in results between subjects, tasks or modalities, and so minimized bias resulting from the selection of the Windows.

### Statistical analyses

Electrodes located over the same areas of the scalp were combined into Channel Groups as follows: Prefrontal - Fp: Fp1 Fp2, Frontal - F: F3 F4, Central - C: C3, C4, Vertex - Cz, Temporal - T: T3, T4, Parietal - P: P3, P4, Pz, Occipital - O: O1, O2. The statistical analysis of mean ERSP by Channel Group was carried out by Window with Repeated Measures Analysis of Variance (RM-ANOVA) in a model including 2 factors (Interval and Channel Group). In this model, the Interval factor reflects the effect of time on task (or Sustained Attention) and the Channel Group factor reflects effect of the location of scalp EEG recordings. ERSP induced by electrical stimuli were not analyzed because the Windows may not be comparable to the laser [7]. A Wilcoxon signed rank test was performed for the follow up analysis of differences between Channels (‘anovan.m’ in Mat lab).

ERSP was also analyzed with respect to Errors by a RM-ANOVA, including factors of Channel Group, and Errors. The Error factor consisted of two levels, the eight subjects with the highest Error Rates and the eight with the lowest Rates.

Other statistical analyses were carried out using standard techniques [25]. The Window IV Beta ERS was correlated with Errors as a Primary Outcome, and the other Windows were correlated with Errors as Secondary Outcomes. Correlations were assessed for Errors versus ratings and versus mean ERSP of individual Channel Groups by linear regression across patients (Mat lab, ‘corrcoef.m’).

## Results

### ERSP spectral windows

Analyses were carried out in the time-frequency domain over time on task while subjects counted the Laser pulses. Figures 1A and B show ensemble time-frequency plots for the First and Second Intervals averaged across Subjects and Channels. Windowing parameters for laser stimuli are shown in Table 1, and the approximate location for Windows in time frequency plots is indicated by roman numerals I to IV in Figures 1A and 2B.

Time-frequency plots for both Intervals showed Delta/Theta early and later components (Windows I and II, Table 1). These plots also show an Alpha ERD which decreased in the Second Interval (Window III, Figure 1) followed by a Beta rebound ERS which increased in the Second Interval (Window IV). The time frequency Windows I to IV (Table 1) for the oscillatory activities following the laser are shown in Figure 1A and are similar to those reported in previous studies of activity induced by the laser stimulus [7,26,27].

### ERSP related to interval and channel

In this section, we examine ERSP by Window using a model with factors of Interval and Channel Group. In Windows I and II the Channel Group factor was significant (ANOVA, *F*_6,105_ =7.45, p<0.00001, and *F*_6,105_ = 5.68, P=0.0005) but there was no significance for the Interval Factor or the Interaction Term. Followup testing did not show that significant differences in ERS by Channel for Window I or II. Therefore Windows I and II showed that ERSP was related to Channel Group factor alone.

In Window III the Interval factor was significant (*F*_6,105_ = 4.54, P=0.034) but not the Channel Group factor or Interaction Term. Testing between channels showed greater ERD in the Second Interval of time on task for P4 (P=0.023, Wilcoxon) and Pz (P=0.02) [25].

In Window IV, significance was found for the Interval factor (*F*_6,105_ = 4.1, P=0.044) while the Channel Group factor and Interaction Term were not significant. Testing between channels showed greater ERS in the Second Interval for P4 (P=0.03, Wilcoxon). These results are shown in Figure 1 by the decrease in ERD (cold colors) for Window III and the increase in ERS (hot colors) for Window IV with time on task. In Figures 1 and 2 ERS (Windows I, II and IV) was defined by ERSP larger than 1 (Hot colors see Methods), and ERD was defined by ERSP less than 1 (Window III, Cold colors), where both were displayed relative to thresholds for significance (Green).

In summary, Window III showed decreased ERD and Window IV showed increased ERS in the second Interval (Alpha Beta Sequence), which reflects the effect of time on task or Sustained Attention upon the response to the painful laser. Only three channels over these Windows showed a significant change in ERSP by Interval and two of these occurred at the same Channel (P4) which is unlikely to occur at random (P<0.0002, combinatorial).

### Ratings, errors and ERSP

The Error Rates for the laser were higher in the Second Interval than the First Interval in more subjects than expected at random (12/16, P=0.027 Binomial), which is consistent with the Vigilance Decrement as in other modalities [28-30]. It has been suggested that errors in a CPT are dependent upon the characteristics of the target stimulus, particularly the magnitude and salience of the evoked sensation [19,31]. Therefore, we tested the correlation between Error Rates in the Second Interval versus laser energy, and ratings of pain and salience across subjects. Errors were inversely related to the ratings of salience (R= −0.51, P=0.045) and unpleasantness of the laser pulse (R=−0.52, P=0.044), and to the laser energy (R= −0.53, P=0.040). These results demonstrate that characteristics of Sustained Attention to other modalities are also found to pain including the Sustained Attention decrement, and Vigilances were more frequent for stronger stimuli which were rated as more unpleasant and salient.

We display time frequency plots for two groups of subjects: the eight with the highest Error Rates and the eight with the lowest Rates (Figures 2A and B). To test our Primary Outcome of an association between Errors and Beta band activity, we carried out an ANOVA on Window IV ERS by factors of Errors and Channel Group. The Error factor was significant (*F*_1,110_ = 3.97, P=0.043) but not the Channel Group or Interaction Term. Window IV ERS in the subjects with the High Error Rates was greater than those with Low Rates and is in keeping with our hypothesis.

As secondary outcomes, we examined Errors in the other Windows (I, II and III) and same Channel Groups. In both Windows I and II (Delta/Theta), the Error factor (*F*_1,110_ = 9.15, P=0.0032 and *F*_1,110_ = 6.93, P=0.009) and Channel Group factor (*F*_1,110_ = 7.14, P=0.00001 and *F*_1,110_ = 4.97, P=0.0002) were significant but not for the Interaction Term. The Error factor by subject is shown in Figure 2 by the increased Window I ERS, which is absent in the High Error subjects. The situation was reversed for Window II ERS which was greater in the subjects with the High Error Rates. In the case of Window III ERD neither the factors nor the Interaction was significant. In summary, High Error Rates were associated with higher ERS for Beta (Window IV) and late Delta/Theta (Window II), and with lower ERS for early Delta/Theta (Window I).

The Primary Outcome was also tested by linear regression of Error Rates in the Second Interval upon the Beta Window IV in each Channel Group. A significant correlation between Window IV and Errors was found for Central Channels (R=0.54, P=0.03, Figure 2C). Testing of Secondary Outcomes revealed that Error Rates were correlated with Prefrontal Delta/Theta ERS (R=0.62, P=0.01). These results suggest that Beta ERS is related to Sustained Attention to pain and to the associated Errors.

## Discussion

We have tested the hypothesis that painful target stimuli during a CPT will induce decreases in ERSP with time on task across Windows, while Beta ERSP will correlate with Errors in the CPT. Substantial changes in ERSP were found for Windows III and IV, and the Alpha Beta Sequence was related to Sustained Attention toward the painful laser stimulus. ERD in the Alpha band and ERS in the Beta band have been previously been reported in response to painful stimuli and with attention to painful stimuli [8,26,32-35]. These and the present report are consistent with ERSP in these bands being related to different functions, tasks, and behaviors [36-38].

Imaging studies suggest that there are changes in frontal and parietal hemodynamic activations during Sustained Attention to auditory, visual, and nonpainful somatic stimuli, and that these activations can increase, decrease or be unchanged with time on task [20,21,23]. None of the stimuli in these studies is unpleasant or painful, and the imaging analyses are very different from analysis of EEG. Nevertheless, Windows III and IV show opposite changes in ERSP with time on task which might explain the disparate imaging results, and Window IV Beta ERS is greater in subjects with high Error Rates.

### An ERSP sequence for sustained attention to pain

We report a Sequence of decreased Alpha ERD (Window III) followed by increased Beta ERS (Window IV) with Sustained Attention to a painful stimulus. Sensory stimuli often lead to excitatory responses followed by inhibition of cortical neurons [39,40], which are mediated in part by thalamocortical pathways [40,41]. A decrease and rebound of Beta activity are observed after painful [42.^43^] and nonpainful somatic sensory stimuli [44,45]. Therefore, the present decrease in Alpha followed by an increase in Beta may represent decreased excitation followed by increased inhibition [46,47].

A Sequence of increased Alpha and increased Beta has been reported over central structures during attention to phasic painful stimuli in an oddball protocol [26,27] and nonpainful somatic sensory stimuli [44,45]. None of these studies employed an experimental Sustained Attention protocol or a painful stimulus and so would not be directly comparable to the present study.

Window III has decreased ERD with time on task, which might be attributed to habituation - the decrease in neural responses over time in a train of stimuli. Habituation of EEG activity has been studied in protocols with prolonged trains of painful stimuli and a task of rating stimuli overall at the end of the train. Protocols of this type have produced decreases in the Alpha and Beta band ERSP with time during a train of painful stimuli [48,49], while increases in ERSP were not reported in any frequency band, including Beta. Although their stimulus trains were prolonged, none of the habituation protocols included a task directed component, which is further evidence that the present Alpha Beta Sequence may result from Sustained Attention toward pain. The present protocol produces Sustained Attention reflecting task directed, and stimulus driven processes that result from the random timing and ordering of two modalities of stimuli in the same train [50,51].

### Errors during sustained attention to pain

During Sustained Attention to nonpainful modalities, activity recorded over parietal or central structures may also be related to errors in tasks across a number of modalities. For example, decreases of post event Beta have been associated with errors occurring during visual attention [11], or in the execution of movements [52]. In the former case, correct responses were associated with increased Beta synchrony in structures including bilateral parietal and frontal, as well as cingulate structures. In the present CPT, Errors were a factor in post event Beta ERS, and were correlated with Beta activity at Central Channels.

Errors during Sustained Attention to visual stimuli are associated with altered activity in structures including parietal cortex, while recovery following an Error is associated with increased activity in the inferior frontal gyrus and parietal cortex [53]. In turn, lesions of parietal cortex are associated with errors of detection [54], and with errors associated with neglect to multiple modalities including pain [55,56]. Together with the present results, these reports suggest that parietal cortex is a focus for Sustained Attention, and possibly for hypervigilance to painful stimuli [1].

Among healthy subjects during a CPT with intense but nonpainful targets, increases in errors were correlated with the unpleasantness of the target [12]. In another study, errors in a visual Sustained Attention and working memory task were correlated with pain of any etiology at the time of the online testing in a large internet survey in selfenrolled individuals [13]. These results may be congruent with those in patients with chronic pain due to fibromyalgia who showed increased errors versus healthy controls [15,57], and correlation of errors with both clinical symptoms and effects of EEC biofeedback therapy [14]. Patients with chronic ‘nonmalignant’ pain versus controls had lower performance and increased errors in a visual CPT task [16]. On this basis, changes in Beta activity with time on task and with Errors in that task are novel evidence of neural activity mediating the effect of Sustained Attention upon pain. Finally, these results suggest a testable hypothesis that parietal cortex may be a locus for anatomically targeted treatment of ‘nociceptive’ pain syndromes, which demonstrate evidence of increased or pathologic Sustained Attention toward painful stimuli [2,58].

## Figures and Tables

**Table 1.** Windows I through IV as defined by poststimulus time range and frequency range.

Laser windows	Time post stimulus	Frequency range
I delta-theta ERS	200 to 500 ms	0 to 8 Hz
II delta-theta ERS	600 to 1400	0 to 8
III alpha ERS	600 to 1200	8 to 14
IV beta ERS	1000 to 1600	14 to 30
